# Bioinformatics-based study on the regulatory network of lipid metabolism-related genes and mechanisms in coronary heart disease

**DOI:** 10.1186/s41065-025-00603-4

**Published:** 2025-11-24

**Authors:** Zunxiong Xiao, Liping Wang, Haoqing Shao, Xiaoying Tian, Qinfang Zheng, Xudong Li

**Affiliations:** 1 College of Traditional Chinese Medicine, Hunan University of Medicine, No. 492 Jinxi South Road, Hecheng District, Huaihua, Hunan Province 418000 China; 2 The First Affiliated Hospital of Hunan University of Medicine, Huaihua, Hunan Province 418000 China; 3 College of Rehabilitation Medicine and Health, Hunan University of Medicine, Huaihua, Hunan Province 418000 China

**Keywords:** Coronary heart disease, Lipid metabolism, WGCNA, LASSO model, Diagnostic biomarkers

## Abstract

Coronary heart disease (CHD), is a complex cardiovascular disease driven by atherosclerosis, resulting from a dynamic interplay between dysregulated lipid metabolism and chronic inflammation. This study integrates bioinformatics analysis of GEO datasets with experimental validation to dissect molecular mechanisms underlying CHD pathogenesis. A total of 487 differentially expressed genes (DEGs) were identified (including 295 upregulated and 192 downregulated), with hub genes such as *CD36*, *ALDH2*, *TNF-α*, and *IL1B* highlighted in lipid handling, oxidative stress, and pro-inflammatory cascades. Weighted gene co-expression network analysis (WGCNA) revealed aberrant activation of lipid metabolism-related modules in CHD patients. KEGG enrichment highlighted their involvement in fatty acid transport, cholesterol homeostasis, NF-κB, and the IL-17 signaling. LASSO regression, applied to the combined datasets, identified SERPINA1, and GLUL as diagnostic biomarkers, with in vitro models supporting their pro-atherogenic roles in oxLDL-induced endothelial injury. Animal experiments further validated these findings: CHD rat models exhibited marked upregulation of SERPINA1, and GLUL in myocardial tissue, paralleled by increased M1 macrophage infiltration. Together, this study delineates the intricate lipid-immune axis in CHD and proposes novel candidate biomarkers and therapeutic targets, underscoring their potential for advancing precision medicine in CHD.

**Supplementary Information:**

The online version contains supplementary material available at 10.1186/s41065-025-00603-4.

## Introduction

Coronary heart disease (CHD), a multifactorial and polygenic condition characterized by atherosclerosis, is the underlying cause of acute myocardial infarction (AMI) [1, 2]. The progression from CHD to AMI typically occurs through acute plaque rupture, which triggers thrombosis, leading to coronary occlusion and subsequent myocardial necrosis [3, 4]. Shared risk factors such as chronic inflammation and insulin resistance drive this pathological process [5–7]. As the most severe acute complication of CHD, AMI can be anticipated using modern imaging techniques that detect high-risk plaques [8, 9]. Therefore, effective prevention and management of CHD are essential to reduce the incidence of AMI and improve patient outcomes.

Dysregulation of lipid metabolism is a key driver of CHD, manifested primarily as cholesterol accumulation, imbalance in lipid species, and related inflammatory responses [10, 11]. Studies have demonstrated that the aberrant expression of lipid metabolism-related genes significantly contributes to CHD pathogenesis [12, 13]. Moreover, abnormal lipid metabolism in epicardial adipose tissue (EAT) is positively correlated with the severity of CHD [14]. However, the multifactorial and polygenic nature of CHD limits the effectiveness of traditional experimental approaches. The emergence of bioinformatics has facilitated integrative omics analyses, providing new insights into the molecular mechanisms of CHD.

The progression of CHD is closely associated with dynamics in lipid metabolism, particularly disturbances in low-density lipoprotein cholesterol (LDL-C), high-density lipoprotein cholesterol (HDL-C), and triglycerides (TGs) [15]. Excessive LDL-C promotes vascular lipid deposition, endothelial dysfunction, inflammation, and foam cell formation, accelerating atherosclerosis [16]. In contrast, reduced HDL-C impairs reverse cholesterol transport and worsens atherosclerotic cardiovascular disease (ASCVD) [17]. Lipid metabolism-related genes are significantly enriched in cholesterol metabolic pathways, PPAR signaling pathway, and inflammatory cascades [18]. For example, PPARα is a key transcription factor involved in regulating fatty acid metabolism and cholesterol transport, thereby influencing CHD progression [19]. Acting as a pro-inflammatory mediator and apoptosis-inducing factor, ceramide directly mediates the transition from dysregulated lipid metabolism to cardiovascular pathology, serving as a critical molecular bridge between these processes [20]. Meanwhile, inflammatory cytokines such as IL-1β accelerate atherosclerotic progression by promoting lipid oxidation and foam cell formation [21]. Elucidating the expression and regulatory mechanisms of these genes is essential for understanding CHD pathogenesis and identifying key biomarkers related to lipid metabolism.

Recent advances in single-cell multi-omics technologies have elucidated cellular heterogeneity and key regulatory networks in CHD by integrating transcriptomic and epigenomic data [22]. Spatial multi-omics integrated with imaging techniques enables precise spatial mapping of molecular events within atherosclerotic plaques, guiding targeted interventions [23]. Multi-cohort analyses utilizing datasets such as GSE12288 have identified immune-regulatory genes associated with HDL-C/MHR ratios [24], while metabolome-histone modification studies revealed mechanisms of epigenetic-metabolic dysregulation [25]. Machine learning models, particularly random forest algorithms trained on datasets like GSE202626 significantly improve CAD diagnostic accuracy, offering novel tools for precision medicine [26].

This study utilizes bioinformatics to systematically unravel the regulatory networks of lipid metabolism genes in CHD, supported by in vitro cellular assays and animal experiments for functional validation. Ultimately, this study aims to establish theoretical foundations and identify therapeutic targets for precision medicine in CHD.

## Materials and methods

### Data acquisition and preprocessing

This study retrieved CHD-related gene expression datasets from the Gene Expression Omnibus (GEO) database, specifically the GSE66360 dataset on the GPL570 platform, which includes 13 normal samples and 21 acute myocardial infarction samples, with detailed information provided in Table S1. The downloaded data were normalized via the R package “limma” (v3.56.2) [27] to ensure accurate analysis through background correction and data normalization.

### WGCNA analysis

Weighted Gene Co-expression Network Analysis (WGCNA), implemented via the WGCNA R package (v1.72), was utilized to identify functionally significant gene modules correlated with CHD pathogenesis [28]. A sample clustering dendrogram is provided in Fig. S1. A correlation analysis of gene expression levels across samples was used to construct a weighted gene coexpression network. The soft threshold (power = 9) was selected to ensure that the network exhibited a scale-free topology (scale-free R^2^ >0.85). Genes were clustered into modules with similar expression patterns, and modules significantly associated with CHD were selected for further analysis.

### Differential expression analysis

Differentially expressed genes (DEGs) between CHD and control samples were identified via the R package “limma” (v3.56.2). Genes with a *p*-value < 0.05, an absolute log2-fold change (|log2FC| >1) were considered significantly differentially expressed and included in subsequent analyses.

### Screening of lipid metabolism genes

Lipid metabolism-related genes were extracted from the GeneCards (v5.12, https://www.genecards.org/) [29] via the keyword “lipid metabolism”. Genes were rigorously filtered by Score >20 and gifts >50 criteria to prioritize high-confidence candidates. These genes were used for cross-analysis with DEGs to identify key lipid metabolism-related genes in CHD.

### Gene intersection analysis

The intersection of lipid metabolism genes, DEGs, and key module genes from the WGCNA was performed via VENNY 2.1.0.

### KEGG and GO enrichment analysis

Functional enrichment analysis was conducted via the clusterProfiler R package (v4.8.1) to perform Gene Ontology (GO) and Kyoto Encyclopedia of Genes and Genomes (KEGG) pathway enrichment analyses specifically on DEGs. GO analysis included the biological process (BP), cellular component (CC), and molecular function (MF) categories. KEGG analysis was used to explore metabolic and signaling pathways associated with the candidate genes. The results were visualized through bubble and bar plots with a significance threshold of *p* < 0.05 and an FDR < 0.05.

### Protein‒protein interaction (PPI) analysis

To explore protein‒protein interactions among the candidate genes, the STRING (v12.0, https://string-db.org) [30] was used to construct a protein–protein interaction (PPI) network. The species was set to “*Homo sapiens*,” and interactions with a score ≥ 0.4 were included. The interaction network was visualized via Cytoscape (v3.9.1) software after the TSV file was imported from STRING.

### LASSO regression analysis

LASSO regression analysis was performed via the glmnet R package (v4.1–8) [31]. CHD status and gene expression data from GSE66360 were integrated, and a proportional hazards model was built via the least absolute shrinkage and selection operator (LASSO) method. The random seed was set to 123, and the λ value was selected through 10-fold cross-validation, with the lambda.min value prioritized to identify a statistically significant and biologically plausible gene set. Fivefold cross-validation was applied to identify the optimal model, with GSE142008 and GSE179789 (Table S1) used as a validation dataset.

### Cells and cell culture

Human umbilical vein endothelial cells (HUVECs) obtained from Immocell Biotech (Xiamen, China) were cultured in DMEM medium (11965118, Gibco, USA) containing 10% FBS (FSD500, Excell Bio, Suzhou, China) and 1% penicillin-streptomycin solution (C0222, Beyotime, Shanghai, China) at 37 °C, in 5% CO_2_. To establish a HUVECs injury model, cells were treated with ox-LDL at a concentration of 50 µg/mL for a duration of 24 h. To knock down the expression of IL-1β, SERPINA1, and GLUL, the pLKO.1 with shRNA construct vector was selected and synthesized by Genepharma (Suzhou, China). Inoculate HUVECs into a 6-well plate, with 2 × 10^5^ cells per well, and culture until the cell fusion degree reaches 70%. Then use transfection reagents to transfer these plasmids into the cells.

### Quantitative RT-PCR

RNA was isolated from cells using Cell/Tissue Total RNA Isolation Kit V2 (RC112, Beyotime, Shanghai, China), and the concentration and purity of RNA were detected using a Nano 600 (Jiapeng Technology, Shanghai, China). Subsequently, the RNA was reverse transcribed into cDNA using HiScript lll 1 st Strand cDNA Synthesis Kit (R312, Beyotime, Shanghai, China). The PCR reaction was then conducted on the CFX96 Touch 1,855,195 real-time fluorescence quantitative PCR instrument (Bio-Rad, USA), using a Universal SYBR qPCR Master Mix (MQ101, Beyotime, Shanghai, China). All reactions were repeated at least three times. Finally, the results were analyzed using the 2^−ΔΔCq^ method, and the relative levels of *IL1B*, *SERPINA1*, and *GLUL* were calculated.

The primer sequences were: *IL1B* forward, 5’-ATGGCAGAAGTACCTGAGCTCG-3’ and reverse, 5’-ACACAAATTGCATGGTGAAGTCAG-3’; *SERPINA1* forward, 5’-CCTGGGCAA CTATGAAGTGG-3’ and reverse, 5’-TCCATGATGTCCCAGGTAGC-3’; *GLUL* forward, 5’-GCTGTGTGGAGCAACTCAAC-3’ and reverse, 5’-CAGGGTCTGCATTGCTCATA-3’; *GAPDH* forward, 5’-AATGGGCAGCCGTTAGGAAA-3’ and reverse, 5’-GCGCCCAAT ACGACCAAATC-3’. All primers were from Sangon Biotech.

### Viability analysis

Cell viability was detected using a Cell Viability Assay Kit (BA00208, Bioss, Beijing, China). The cells in the logarithmic growth phase were inoculated into 96-well plates at 2000 cells per well and incubated under the conditions of 37 °C and 5% CO_2_ for 4–8 h. After the cells adhered to the wall, they were treated according to the experimental groups, with 6 replicate wells set up in each group. The cells in each group were incubated for 24 h. Two hours before the end of incubation, 10 µL of CCK8 solution was added to each well. After incubation, the OD450 was measured with a microplate reader.

### Animal experiments

SPF-grade male Sprague-Dawley rats (8 weeks old, 240 ± 20 g) were obtained from Hunan SJA Laboratory Animal Co., Ltd., and housed under controlled conditions (21–25 °C, 50–60% humidity, 12 h light/dark cycle) with free access to food and water. All procedures were conducted in accordance with the guidelines approved by the Animal Ethics Committee of Hunan University of Medicine (Approval No. 2025-A10053). After one week of acclimatization, rats were randomly assigned to control and model groups. The CHD model was established as previously described with minor modifications [32]. In brief, model rats (*n* = 5) were fed a high-fat diet containing 3% cholesterol, 0.5% sodium cholate, 0.2% PTU, 5% sugar, 10% lard, and 81.3% standard chow for 8 weeks. They also received daily intragastric administration of PTU (25 mg/kg) and intraperitoneal injections of vitamin D3 at weeks 1 (600,000 IU/kg), 4 (100,000 IU/kg), and 6 (100,000 IU/kg). During the 8th week, isoproterenol (1 mg/kg) was administered subcutaneously every other day. Control animals (*n* = 5) received standard chow and equivalent volumes of distilled water or saline.

### Cardiac ultrasound

Rats were anesthetized, and left chest hair was removed. A high-frequency matrix probe was placed perpendicular to the left chest wall to capture left ventricular long-axis and short-axis images along the mitral valve-to-apex direction. Measured parameters included: Left ventricular ejection fraction (EF), Left ventricular fractional shortening (FS), Cardiac output (CO), and Left ventricular mass (LV Mass).

### HE staining

Myocardial tissues fixed in 4% paraformaldehyde were sectioned (5 μm) after graded ethanol dehydration (75–95%), xylene clarification, and paraffin embedding. Sections were stained with hematoxylin (5 min) and eosin (3 min in 85–95% ethanol), differentiated with 1% hydrochloric acid ethanol, and blued with 0.5% ammonia water. Images were captured using a Leica ImageScope system to evaluate myocardial structure, cell arrangement, interstitial edema, and inflammatory infiltration.

### Western blot

Myocardial tissues were lysed in RIPA buffer containing 1% protease inhibitor cocktail and 1% PMSF. Protein concentrations were determined via BCA assay, and equal amounts (30 µg/lane) were separated by SDS-PAGE (5% stacking gel, 10–12% resolving gel) and transferred to PVDF membranes (300 mA, 90 min). Membranes were blocked with 5% non-fat milk, incubated overnight at 4 °C with primary antibodies: IL-1β (1:3000, Abcam ab283818), SERPINA1 (1:3000, Abcam ab166610), GLUL (1:4000, Abcam ab313449), and β-Actin (1:5000, Abcam ab6276). After TBST washing, HRP-conjugated secondary antibodies (1:5000) were applied for 1 h. Proteins were visualized using ECL and analyzed with Image Lab software.

### Flow cytometry for M1 macrophages

Myocardial tissues were minced and digested with collagenase type I/II at 37 °C for 30–60 min. Single-cell suspensions were filtered (200-mesh), centrifuged (1500 rpm, 5 min), and incubated with CD16/32 antibody (10 min, 4 °C) to block Fc receptors. Cells were stained with FITC-F4/80 (macrophage marker) and APC-CD86 (M1 marker) for 30 min (4 °C, dark). Dead cells were excluded using 7-AAD. Flow cytometry gated FSC/SSC to exclude debris, and M1 macrophages were identified as CD86^+^ F4/80^+^ double-positive cells. Data were analyzed using FlowJo software.

### Statistical analysis

All bioinformatics analyses were performed in R (v4.2.1). Batch effects were corrected using the ComBat method from the sva package. Differential expression analysis was conducted using the limma package with empirical Bayes moderation. P-values were adjusted using the Benjamini–Hochberg method to control the false discovery rate (FDR < 0.05). Genes with |log_2_FC| >1 and *p* < 0.05 were considered significant. In WGCNA, a soft threshold (power = 9) was applied to construct a scale-free network. Module–trait correlations were tested using Pearson correlation and corrected via Bonferroni method. Functional enrichment (GO and KEGG) was used clusterProfiler with FDR correction. LASSO regression was implemented using glmnet with five-fold cross-validation. For the experimentally validated analysis, data were analyzed using GraphPad Prism 9 and are reported as mean ± S.D. Differences between two groups were assessed by an unpaired Student’s t-test, while comparisons across multiple groups were performed using a one-way ANOVA followed by Tukey’s post hoc test. A two-sided p-value of less than 0.05 was considered statistically significant.

## Results

### Results of WGCNA

**Fig. 1:**
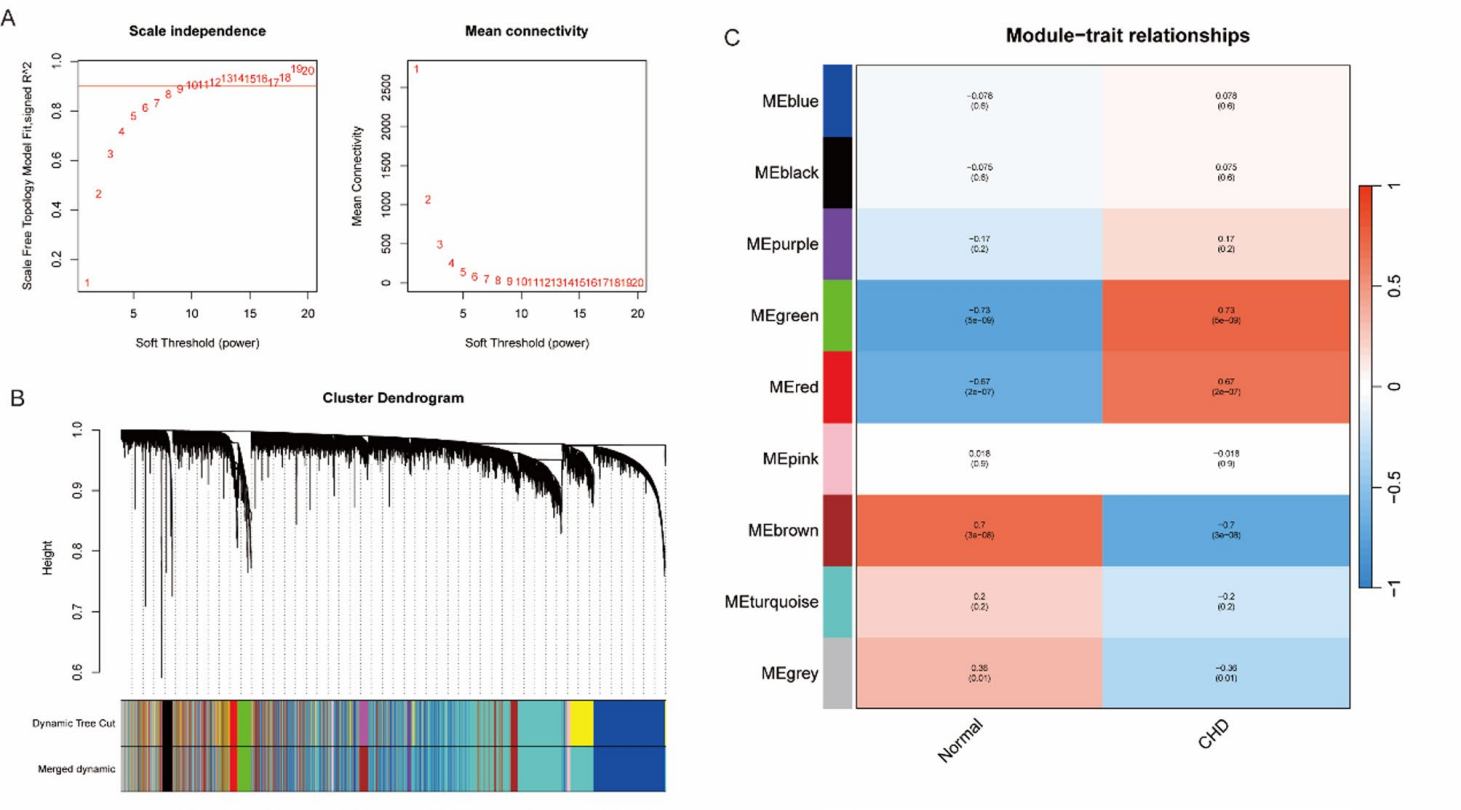
Co-expression network construction and module analysis in CHD. **A** Soft-thresholding selection plots: left panel shows the scale-free fit index (R^2^) across soft-thresholding powers (β); right panel displays mean connectivity. The optimal β = 9 achieved scale-free topology (R^2^ > 0.9) with balanced connectivity. **B** Cluster dendrogram of co-expression modules. Genes are grouped into color-coded modules based on expression similarity. The lower bar indicates dynamic tree cutting and merged modules. **C** Module–trait correlation heatmap between gene modules and clinical traits (normal vs. CHD). Red and blue reflect positive and negative Pearson correlations, respectively; values include r (with *p*-value)

WGCNA was used to construct a co-expression network to identify gene modules associated with CHD progression. A soft threshold of β = 9 was applied to achieve scale-free topology (R^2^ > 0.9) and maintain adequate mean connectivity, ensuring network stability and biological interpretability (Fig. 1A). Hierarchical clustering grouped genes into nine distinct modules, each comprising co-expressed genes with similar expression profiles (Fig. 1B). Module-trait relationship analysis demonstrated significant correlations with CHD, with the green module showing the strongest positive association (*r* = 0.73, *p* < 0.05), which was positively correlated with disease status (Fig. 1C). These findings suggest that genes within this module may contribute to CHD pathogenesis. Overall, the module-based approach offers valuable insights into the molecular mechanisms of CHD and highlights potential biomarkers for further diagnostic and therapeutic investigation.

### Differential expression analysis

**Fig. 2:**
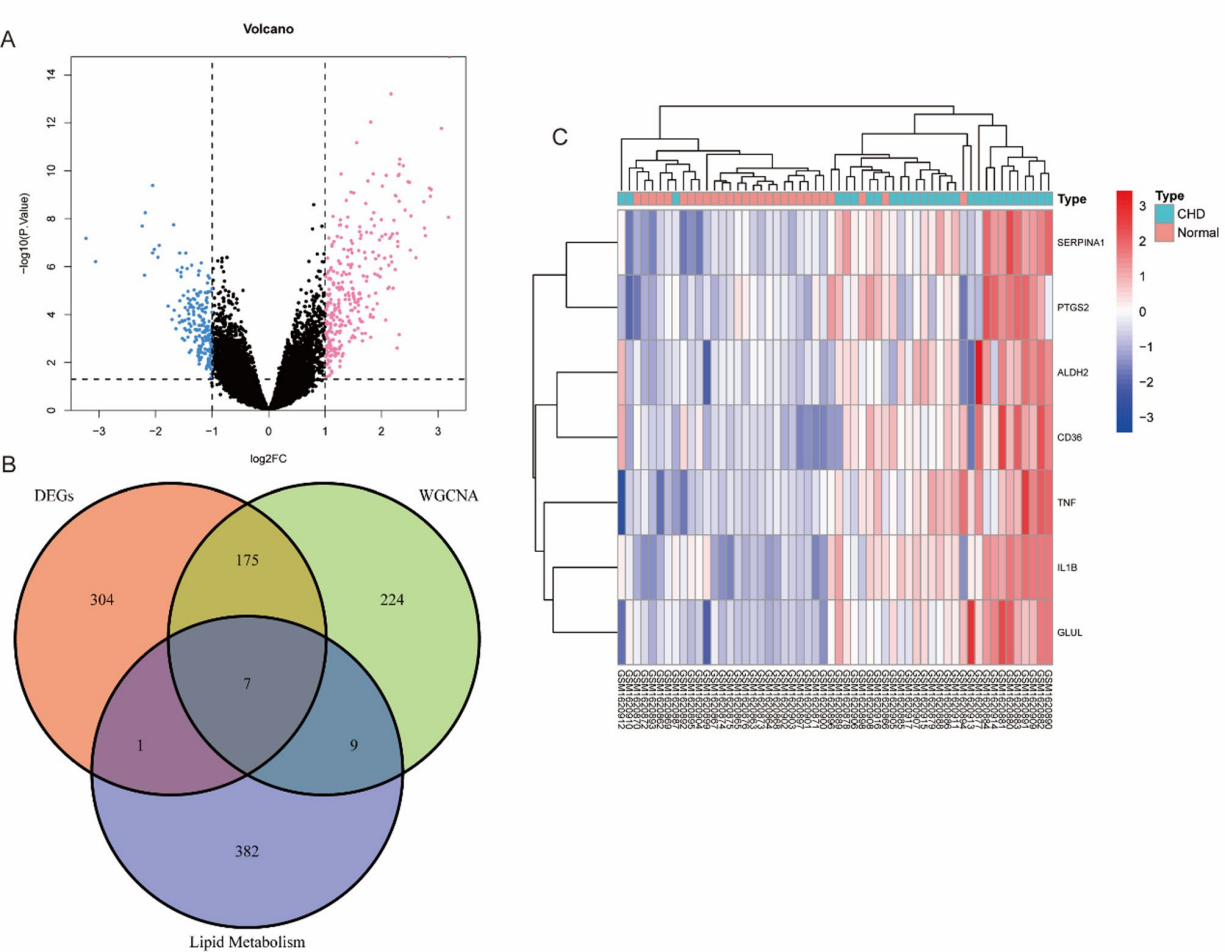
Integrated analysis of CHD-related genes. **A** Volcano plot of differentially expressed genes (DEGs). The x-axis represents log_2_FC; the y-axis shows -log_10_(*p*-value). Significantly downregulated and upregulated genes (*p* < 0.05) are depicted in blue and red, respectively; non-significant genes are shown in black. **B** Venn diagram showing the intersection among DEGs, WGCNA module genes, and lipid metabolism-related genes, revealing seven hub genes implicated in lipid dysregulation in CHD. **C** Heatmap of expression patterns of the seven hub genes in CHD (*n* = 21) and normal (*n* = 13) samples. Red and blue indicate high and low expression, respectively; hierarchical clustering distinguishes CHD from control samples

Differential expression analysis identified 487 DEGs, including 295 upregulated and 192 downregulated genes, as visualized in the volcano plot (Fig. 2A). The volcano plot highlights both the statistical significance and fold changes of gene expression, with significantly upregulated (pink) and downregulated (blue) genes clearly separated from non-significant genes (central cluster). The Venn diagram (Fig. 2B) further shows the overlap between DEGs, WGCNA-derived module genes, and lipid metabolism-associated genes, revealing seven hub genes: *SERPINA1*, *PTGS2*, *ALDH2*, *CD36*, *TNF*, *IL1B*, and *GLUL*. These genes are involved in lipid metabolic dysregulation and chronic inflammation, and likely play pivotal roles in CHD pathogenesis.

Heatmap analysis (Fig. 2C) reveals distinct expression patterns of these hub genes in CHD compared to control samples. Proinflammatory factors such as TNF and IL1B were marked upregulated in CHD, emphasizing their contribution to disease mechanisms. Altered expression of lipid metabolism regulators like *CD36* and *ALDH2* further underscores the importance of metabolic imbalance in CHD progression. Together, these results provide important insights into the molecular basis of CHD and point to promising biomarkers for diagnostic and therapeutic applications.

### Results of the KEGG pathway and GO enrichment analyses

**Fig. 3:**
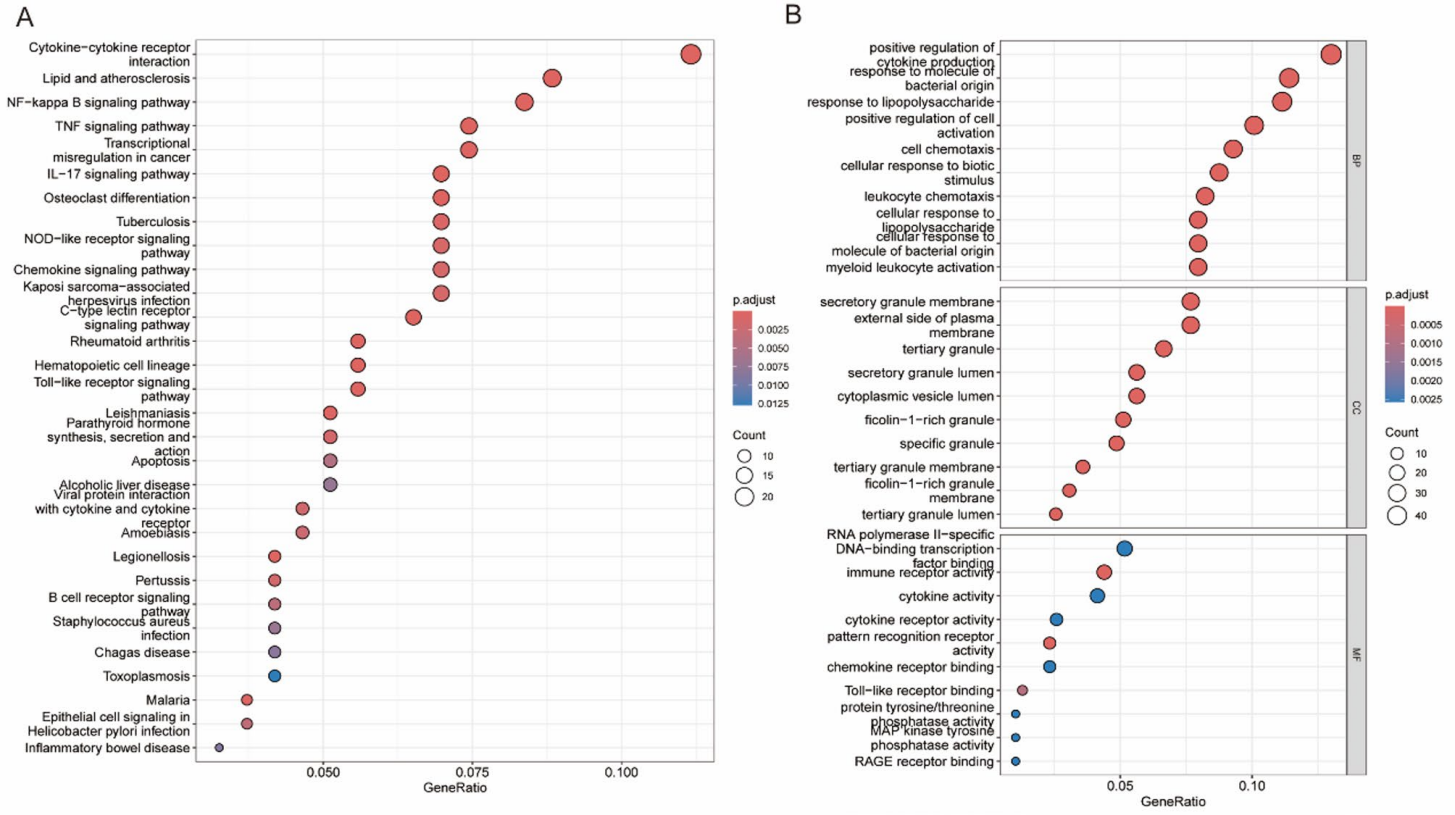
KEGG and GO enrichment analyses in CHD. (A) KEGG pathway enrichment. GeneRatio represents the proportion of genes associated with each pathway; circle size corresponds to gene count, and color intensity (blue to red) indicates statistical significance based on adjusted p-value. Key pathways such as cytokine–cytokine receptor interaction, NF-κB signaling, and IL-17 signaling are highlighted, which are closely associated with inflammatory responses in CHD. (B) GO enrichment across three categories: Biological Process (BP) terms were enriched in bacterial response and immune activation; CC included secretory granules and vesicles; MF featured cytokine binding and pattern recognition activity. These findings associate DEGs with inflammatory and immune dysregulation mechanisms central to CHD pathogenesis

KEGG and GO enrichment analyses were performed on DEGs to elucidate the key pathways and biological processes involved in CHD pathogenesis. KEGG analysis (Fig. 3A) indicated significant enrichment of inflammatory and immune-related pathways, with cytokine-cytokine receptor interaction pathway exhibiting the highest gene ratio. Other prominent pathways included lipid metabolism and atherosclerosis metabolism, NF‒κB signaling, TNF signaling, and IL‒17 signaling, which are closely associated with chronic inflammation and immune dysregulation. Notably, several infection-related pathways such as tuberculosis and *Staphylococcus aureus* infection were also enriched, implying a potential link between microbial infection and CHD-related immune responses.

GO analysis (Fig. 3B) provided further mechanistic insights across three categories: (1) Biological Processes (BP): including response to bacterial molecules, chemokine-mediated signaling, and immune cell activation, underscoring pathogen-driven inflammation (2). Cellular Components (CC): such as secretory granules and vesicle lumens, indicative of inflammatory mediator storage (3. Molecular Functions (MF): including cytokine binding and Toll-like receptor activity. These results collectively highlight the interplay between dysregulated lipid metabolism, chronic inflammation, and immune dysfunction in CHD, offering a systematic perspective on potential therapeutic targets for modulating inflammatory pathways.

### ROC curve and protein‒protein interaction network analysis

**Fig. 4:**
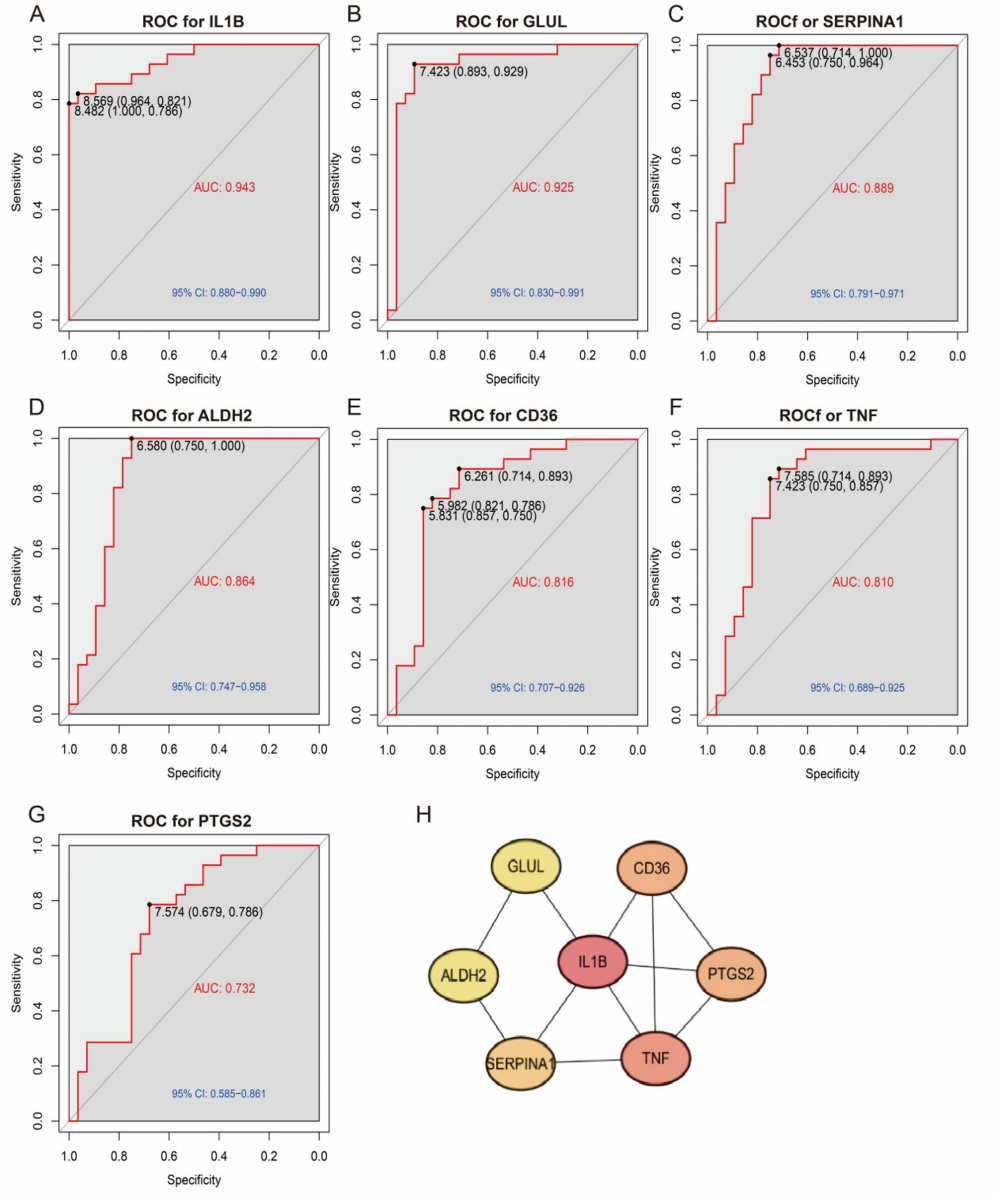
ROC curves and PPI network analysis of key differentially expressed genes. **A**‒**G** ROC curves based on the GSE142008 dataset for seven hub genes.Respective AUC values: IL1B (0.943, 95% CI: 0.880–0.990), GLUL (0.925, 95% CI: 0.830–0.991), SERPINA1 (0.889, 95% CI: 0.791–0.971), ALDH2 (0.864, 95% CI: 0.830–0.991), CD36 (0.816, 95% CI: 0.707–0.926), TNF (0.810, 95% CI: 0.689–0.925), PTGS2 (0.732, 95% CI: 0.585–0.861). Specificity (x-axis); Sensitivity (y-axis). Higher AUC indicates greater diagnostic accuracy. **H** PPI network with IL-1β as a central node, interacting with TNF, CD36, and PTGS2. Node color indicates expression (red: upregulated; blue: downregulated); edges represent functional associations. The network underscores the pivotal role of IL-1β in CHD pathogenesis via inflammatory and metabolic dysregulation

Receiver operating characteristic (ROC) curve analysis, performed using the GSE142008 dataset, demonstrated the diagnostic potential of seven core DEGs (Table S2, Fig. 4A-G). IL1B achieved the highest AUC value of 0.943, demonstrating strong potential as a biomarker for CHD and reflecting its pro-inflammatory role. The high predictive accuracy of GLUL (0.925) and SERPINA1 (0.889) further supports their diagnostic relevance. Specifically, GLUL may contribute to CHD through its involvement in glutamate metabolism and endothelial dysfunction, while SERPINA1 appears to be linked to protease inhibition and inflammation modulation. Moreover, protein–protein interaction (PPI) network analysis highlighted the functional collaboration among IL-1β, TNF, and CD36 in promoting inflammatory and lipid metabolic dysregulation, offering mechanistic insights into CHD pathogenesis. These findings provide a foundation for future biomarker development and therapeutic targeting (Fig. 4H). 

### LASSO regression-based feature gene selection results

**Fig. 5:**
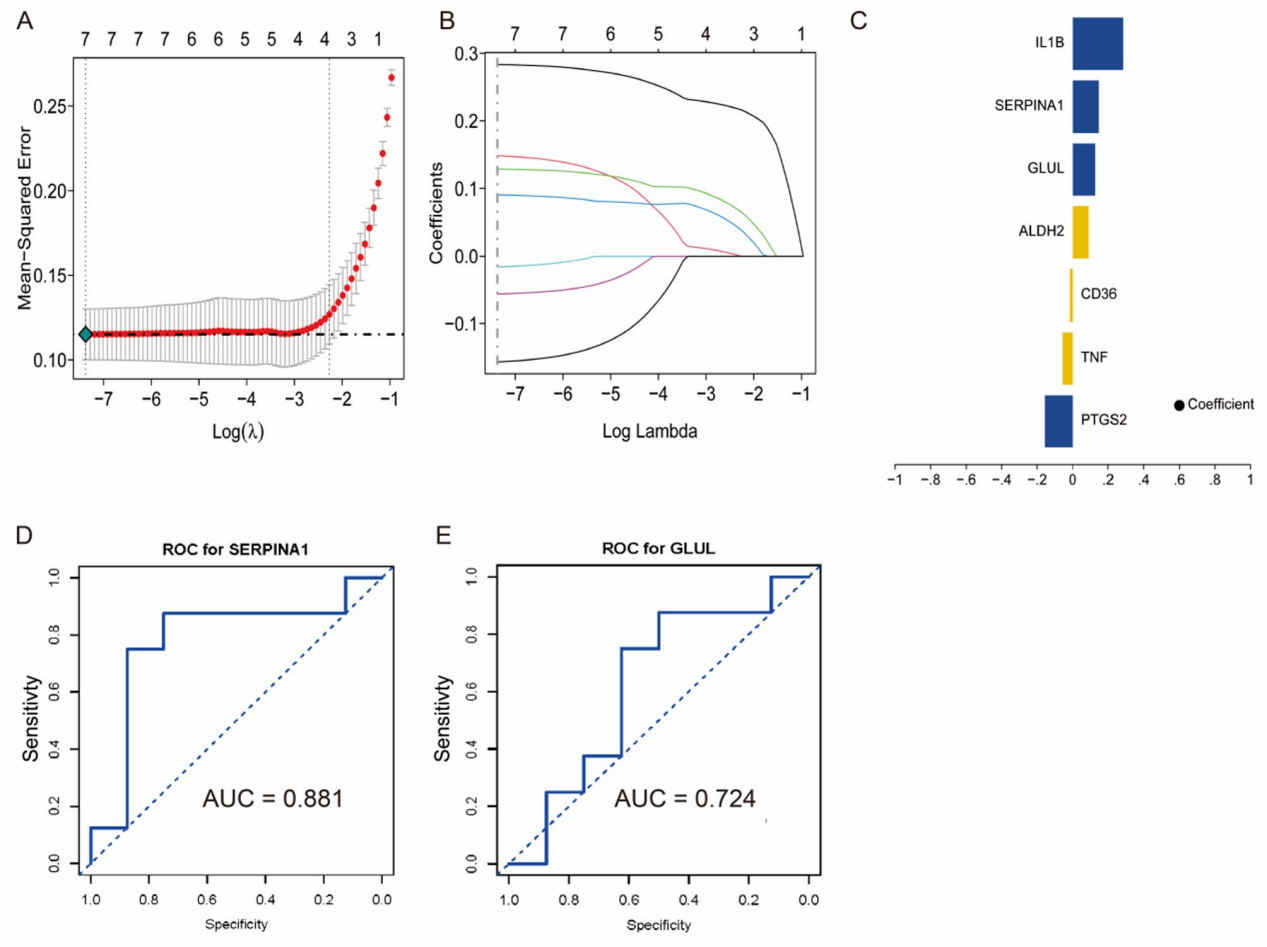
Results of feature gene selection via the LASSO regression model. **A** Cross-validation curve (log(λ) versus MSE) showing selection of the optimal λ value corresponding to the minimum mean squared error. **B** Coefficient profile plot illustrating shrinkage of regression coefficients with increasing λ, where irrelevant genes are driven to zero. **C** Bar plot displaying coefficients of selected genes: blue bars (IL1B, GLUL, SERPINA1) represent positive associations with CHD risk (AUCs: 0.943, 0.889, 0.925), while blue bars (PTGS2, TNF, CD36) suggest protective effects. **D**-**E** ROC analysis using the independent dataset GSE179789 confirms the strong diagnostic performance of SERPINA1 (AUC = 0.881) and GLUL (AUC = 0.724), supporting their utility as biomarkers for CHD

LASSO regression was used to identify predictive gene signatures for CHD. Figure 5A displays the optimal γ value that minimized the mean squared error (MSE), balancing model accuracy and simplicity. The coefficient shrinkage path (Fig. 5B) illustrates how less relevant features were penalized, retaining the most influential genes. Key predictors included IL1B (coefficient = 0.42), SERPINA1 (0.38), and GLUL (0.35), with weaker contributions from PTGS2, TNF, and CD36 (Fig. 5C). External validation using dataset GSE179789 confirmed strong performance, with AUC values of 0.881 for SERPINA1, 0.724 for GLUL (Fig. 5D-E). Regression weights further suggest distinct functional roles: SERPINA1 in inflammation and GLUL in lipid metabolism. These validated biomarkers enhance CHD diagnosis and support personalized treatment strategies, while providing mechanistic insight for future research.

### Functional validation of SERPINA1 and GLUL in a vascular endothelial cell injury model

**Fig. 6:**
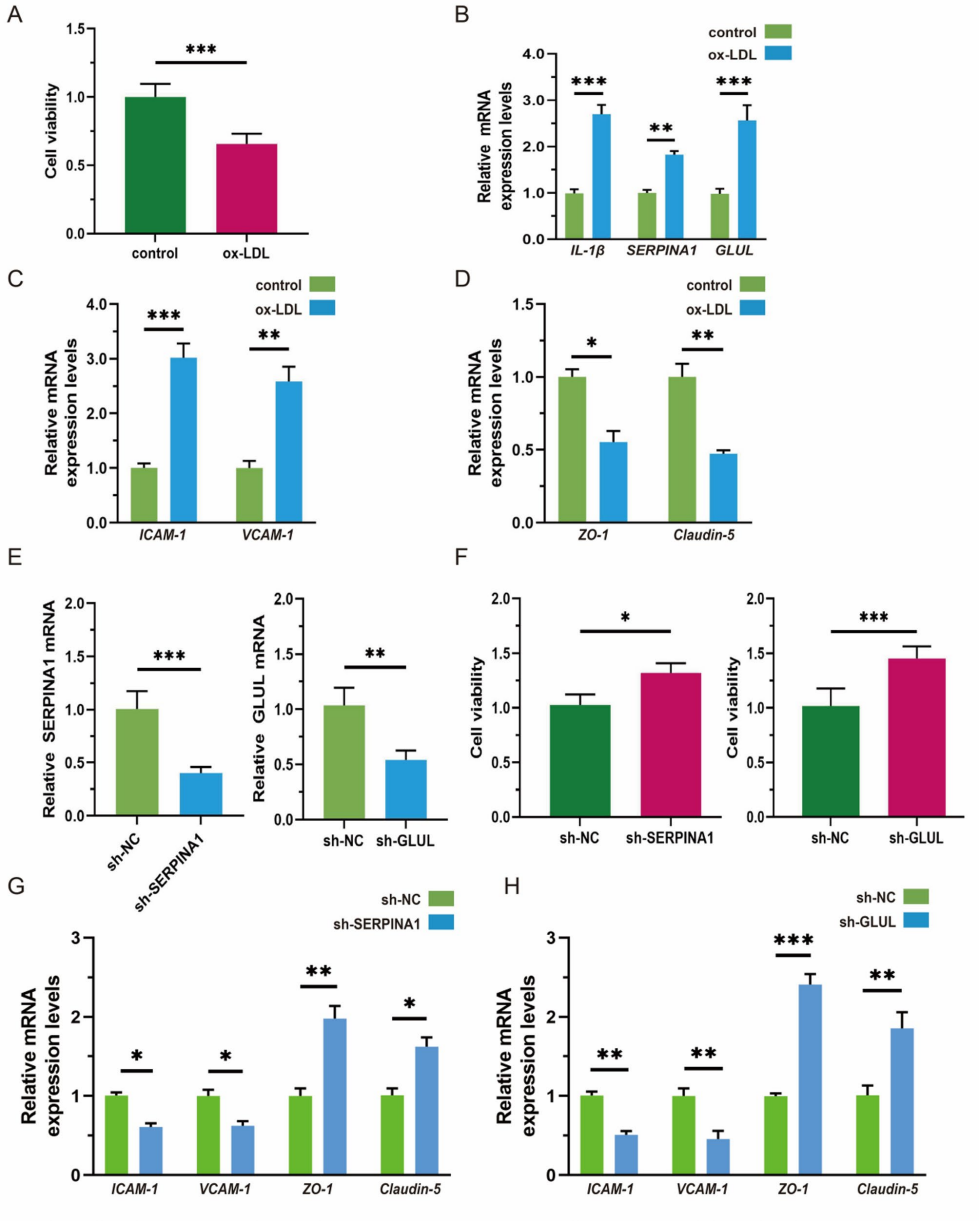
Mechanistic insights into SERPINA1 and GLUL in a vascular endothelial cell injury model. **A** ox-LDL treatment significantly reduced endothelial cell viability. **B** Expression levels of IL1B, SERPINA1, and GLUL were upregulated following ox-LDL exposure. **C** ox-LDL induced elevated expression of endothelial injury markers (ICAM-1, VCAM-1). **D** Endothelial permeability markers (ZO-1, Claudin-5) were downregulated after ox-LDL treatment. **E** Knockdown efficiency of SERPINA1 and GLUL was confirmed. **F** Silencing SERPINA1 and GLUL attenuated ox-LDL-induced endothelial injury. **G**-**H** knockdown of SERPINA1/GLUL, the expression of ICAM-1 and VCAM-1 was decreased, while ZO-1 and Claudin-5 was increased. The results are expressed as mean ± SEM. *n* = 6 for each group. ^*^p < 0.05, ^**^p < 0.01,^***^p < 0.001

To elucidate the roles of SERPINA1 and GLUL in endothelial injury, an in vitro model was established using human umbilical vein endothelial cells (HUVECs) treated with 50 µg/mL ox-LDL for 24 h. CCK-8 assays showed that ox-LDL significantly reduced cell viability compared to the control (Fig. 6A, *P* < 0.001). qRT-PCR analysis revealed pronounced upregulation of *SERPINA1* (*P* < 0.01) and *GLUL* (*P* < 0.001) mRNA expression (Fig. 6B). Furthermore, ox-LDL treatment increased the expression of endothelial injury markers (ICAM-1, VCAM-1; Fig. 6C) and decreased endothelial junction markers (ZO-1, Claudin-5; Fig. 6D), indicating disrupted barrier function. Knockdown of SERPINA1 or GLUL significantly alleviated ox-LDL-induced loss of cell viability (Fig. 6E-F), reduced the expression of endothelial injury markers ICAM-1 and VCAM-1, and up-regulated tight junction proteins ZO-1 and Claudin-5(Fig. 6G-H), indicating a protective effect. These results demonstrate that both SERPINA1 and GLUL contribute to ox-LDL-induced endothelial injury.

### Multidimensional experimental validation of immune microenvironment dysregulation in a CHD rat model

**Fig. 7:**
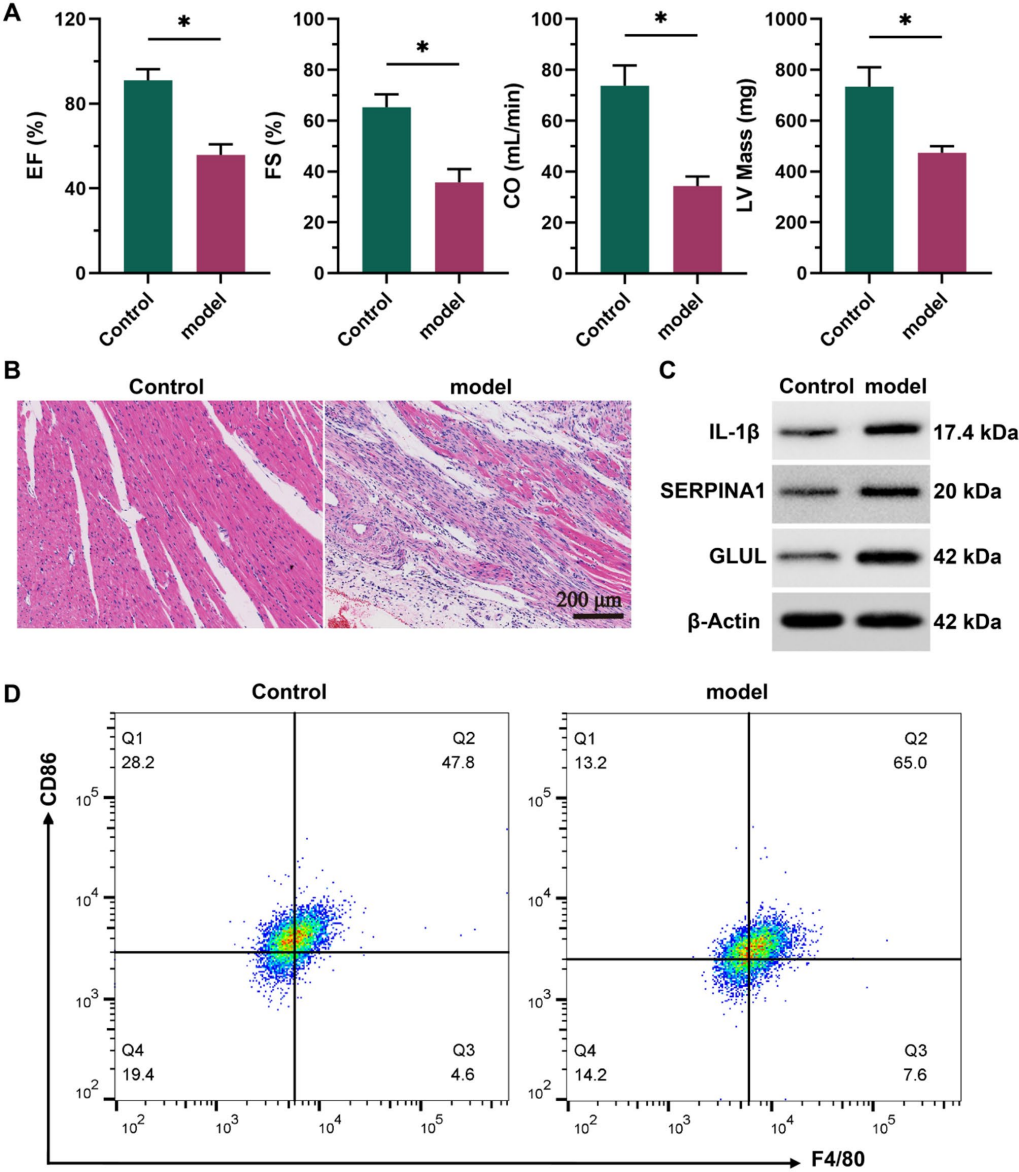
Experimental validation of immunopathological features in a CHD rat model. **A** Echocardiography indicated significantly decreased EF, FS, CO, and LV Mass in the model group compared with controls (^*^p < 0.05), demonstrating impaired cardiac function. **B** HE staining revealed disordered cardiomyocyte arrangement accompanied by necrosis/calcification, interstitial fibrosis, and mild-to-moderate inflammatory infiltration. **C** Western blot showed markedly increased myocardial expression of IL-1β, SERPINA1, and GLUL in the model rats. **D** Flow cytometry detected a significantly elevated proportion of M1 macrophage within myocardial tissue

Following the establishment of a CHD rat model, pathological features were validated through multidimensional experiments. Echocardiography indicated significantly decreased EF, FS, CO, and LV mass in model rats compared with controls (Fig. 7A, *P* < 0.05), reflecting progressive cardiac impairment. HE staining displayed disorganized cardiomyocytes with necrosis and calcification, substantial interstitial fibrosis, and mild-to-moderate inflammatory infiltration (Fig. 7B). Western blot analysis confirmed elevated myocardial expression of IL-1β, SERPINA1, and GLUL (Fig. 7C). Flow cytometry showed a significantly increased proportion of M1 macrophages in myocardial tissue (Fig. 7D). These results align with prior bioinformatics findings, offering comprehensive validation of immune microenvironment dysregulation in CHD across molecular, cellular, and histopathological levels.

## Discussion

This study integrated bioinformatic analysis with experimental validation to investigate the regulation of lipid metabolism-related genes in CHD and identified immune dysregulation as a major contributor to disease progression. Our results show that the abnormal expression of genes such as *CD36* and *ALDH2* is closely associated with the development of atherosclerosis [33, 34]. Moreover, the upregulation of genes, including *IL1B*, *SERPINA1*, and *GLUL*, underscores the intricate crosstalk between inflammation and lipid metabolism, which plays a pivotal role in CHD pathogenesis.

As a fatty acid/cholesterol receptor, CD36 promotes foam cell formation by enhancing oxidized LDL (oxLDL) uptake, aggravating plaque formation and instability [35]. Previous studies have established that CD36-mediated lipid accumulation is crucial for coronary plaque development [36]. In contrast, ALDH2 mitigates oxidative stress by clearing toxic lipid peroxidation products. Reduced ALDH2 activity, such as through the rs671 SNP, is stronglyassociated with increased cardiovascular risk, especially in East Asian populations [37]. Therapeutic strategies aimed at inhibiting CD36 and activating ALH2 may offer dual benefits by ameliorating lipid metabolic disorders and reducing oxidative damage.

Bioinformatic analyses further confirmed significant upregulation of proinflammatory cytokines such as TNF-α and IL-1β in CHD patients, supporting the role of chronic inflammation in disease progression, consistent with findings from Wu et al. [38]. Notably, OxLDL not only facilitates lipid accumulation via CD36 but also stimulates the secretion of inflammatory cytokines including TNF-α, IL-1β, and IL-8. This inflammatory milieu further enhances CD36-mediated oxLDL uptake, forming a positive feedback loop that accelerates foam cell formation [39]. Additionally, IL-1β activates an NF-κB-dependent proinflammatory cascade, promoting vascular inflammation and remodeling through upregulation of adhesion molecules and chemokines [40]. This dual mechanism emphasizes the convergence of metabolic dysfunction (CD36/oxLDL) and inflammatory activation (IL-1β/NF-κB) in CHD progression.

KEGG pathway analysis highlighted the crucial roles of NF-κB and IL-17 signaling in CHD pathogenesis. NF-κB pathway activation upregulates adhesion molecules and inflammatory cytokines, accelerating atherosclerosis. Enrichment of the IL-17 pathway is closely tied to aggravated vascular inflammation [41]. NF-κB acts as a central mediator in the IL-17 signaling, facilitating immune cell recruitment and activation. This study detected significant IL-17 pathway enrichment in vulnerable plaque regions of CHD patients, corroborating KEGG findings [42]. IL-17/NF-κB activation upregulates VCAM-1/ICAM-1 in vascular endothelium, promoting monocyte adhesion and M1 macrophage polarization [43]. Subsequently, M1 macrophages sustain proinflammatory cytokine secretion (e.g., IL-1β, TNF-α) via the TLR4/NF-κB axis, creating an immune microenvironment skewed toward inflammation due to reduced Treg activity, ultimately accelerating plaque rupture and disease progression [44, 45].

Targeting these key pathways may offer novel avenues for precision medicine of CHD. Using the LASSO regression, this study identified IL1B, SERPINA1, and GLUL as potential biomarkers for CHD. Their diagnostic performance was validated via receiver operating characteristic (ROC) curve analysis, with IL1B showing exceptional predictive value, with an area under the curve (AUC) of 0.943. As a key proinflammatory factor in CHD, IL-1β promotes atherosclerosis formation and plaque instability through multiple mechanisms [46]. SERPINA1 reduces the risk of cardiac amyloidosis by inhibiting plasmin-mediated transthyretin hydrolysis and aggregation [47]; it modulates the Eph receptor B2 signaling pathway to improve energy metabolism and glucose homeostasis, thereby influencing cardiometabolic diseases [48]. Its subtype, SERPINA3, mitigates myocardial ischemia-reperfusion injury via lactylation modification [49], yet promotes atherosclerotic plaque progression through NF-κB pathway activation [50]. GLUL colocalizes with macrophages in carotid plaques and regulates plaque vulnerability [51]; it serves as a diagnostic biomarker for atrial fibrillation (AUC = 0.767) and heart failure (AUC = 0.76) [52], and modulates immunometabolism via the TLR4/NF-κB pathway-supplementing α-KG or overexpressing GLUL alleviates inflammatory injury [53, 54].

Although this study reveals a complex interaction between lipid metabolism and immune dysregulation in coronary heart disease, several limitations merit consideration. First, reliance on public GEO databases led to limited sample size and technical heterogeneity, such as differences across microarray platforms, which may introduce batch effects, reduce statistical power, and constrain the generalizability of DEGs and WGCNA modules. Second, bioinformatics approaches including differential gene identification, WGCNA, and KEGG enrichment revealed associations but could not establish causality. For instance, the functional mechanisms of SERPINA1 and GLUL require validation using functional experiments like CRISPR-based gene editing. Therefore, existing cellular and animal models need deeper functional investigation to clarify key gene roles, and large-scale clinical studies remain essential to verify the biomarker potential of these candidates.

Further research should also explore the interactions between lipid metabolism and the immune responses—these interactions appear to be central to the progression of coronary heart disease. In this process, particular emphasis should be placed on elucidating the mechanistic roles of SERPINA1 and GLUL, including how they participate in the lipid metabolic regulatory network and the remodeling of the immune microenvironment. Integrating multi-omics data (such as transcriptomics, metabolomics, and proteomics) will provide a more comprehensive understanding of the mechanisms underlying coronary heart disease and facilitate the development of personalized treatment strategies.

## Conclusion

This study elucidates the intricate interplay between dysregulated lipid metabolis*m* and immune dysfunction in the pathogenesis of CHD. By integrating bioinformatics, we identified CD36, ALDH2, TNF-α, and IL1B as candidate central mediators linking lipid handling, oxidative stress, and pro-inflammatory cascades. Aberrant activation of NF-κB/IL-17 signaling pathways was found to promote vascular inflammation and plaque destabilization. Furthermore, SERPINA1 and GLUL were identified as potential diagnostic biomarkers, demonstrating robust predictive accuracy, with their pathogenic roles corroborated by both in vitro and in vivo studies. These findings enhance the mechanistic understanding of CHD and highlight promising therapeutic targets for future investigation in precision medicine.

## Supplementary Information

Supplementary Material 1.

## Data Availability

The data utilized and examined in this study are available upon reasonable request from the corresponding author.
