# Supplementary material for: Bioinformatics-based study on the regulatory network of lipid metabolism-related genes and mechanisms in coronary heart disease
Source: Hereditas. 2025 Nov 24;162:231. doi: 10.1186/s41065-025-00603-4 (PMC12642059; doi:10.1186/s41065-025-00603-4)
Supplement: Supplementary file 1 — Supplementary Material 1. [file 41065_2025_603_MOESM1_ESM.docx]

**Table S1** Comprehensive details of GSE66360, GSE142008, and GSE179789 datasets

| ID | source | platform | country | sample type | sample size | Organism |
| --- | --- | --- | --- | --- | --- | --- |
| GSE66360 | GEO | GPL570 | USA | Expression profiling by array | AMI patients (n=49) vs healthy cohorts (n=50) | Homo sapiens |
| GSE142008 | GEO | GPL570 | China | Expression profiling by array | 8 CHD patients with paired pre- and post-treatment samples (n=16) | Homo sapiens |
| GSE179789 | GEO | GPL570 | China | Expression profiling by array | 8 CHD patients with paired pre- and post-treatment samples + 8 healthy individuals (n=24) | Homo sapiens |

**Table S2** Log2-fold change (logFC) and *p*.Value of the seven core genes on GSE66360 dataset

| Gene.symbol | *p*.Value | logFC |
| --- | --- | --- |
| IL1B | 1.81e-05 | 3.09 |
| GLUL | 1.06e-05 | 1.63 |
| SERPINA1 | 9.10e-05 | 2.6 |
| ALDH2 | 5.57e-05 | 2.28 |
| CD36 | 3.46e-04 | 2.68 |
| TNF | 2.63e-03 | 1.61 |
| PTGS2 | 1.72e-04 | 2.38 |


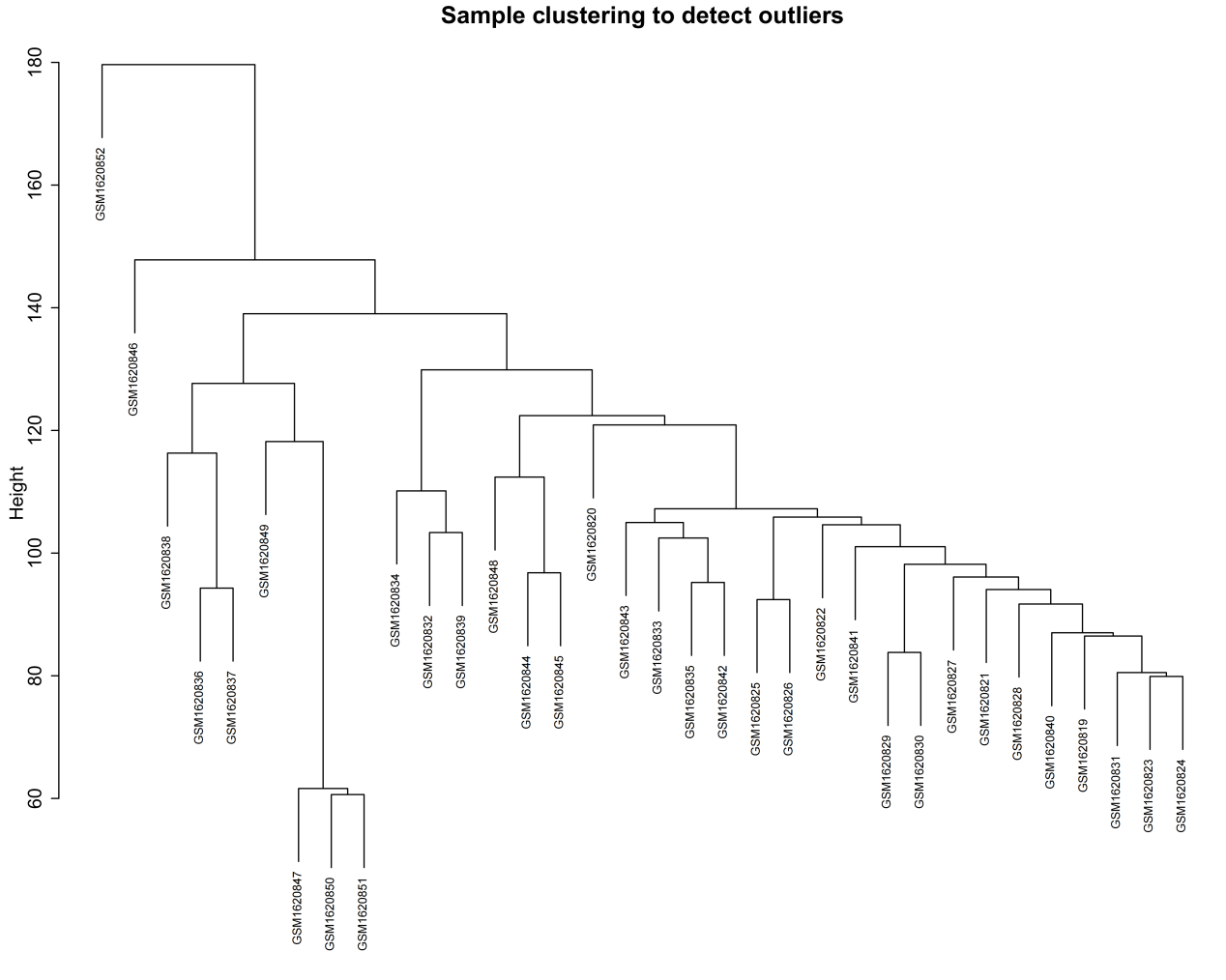


**Fig** **S1** Sample Clustering Plot of GSE66360 (detect outliers). The vertical axis "Height" reflects differences in sample distances, with sample labels (e.g., GSM1123472) marked at the ends. Hierarchical clustering branches, formed by vertical/horizontal lines, display sample clustering relationships and aid in identifying anomalous samples with significant differences.
